# MIMIC-CXR, a de-identified publicly available database of chest radiographs with free-text reports

**DOI:** 10.1038/s41597-019-0322-0

**Published:** 2019-12-12

**Authors:** Alistair E. W. Johnson, Tom J. Pollard, Seth J. Berkowitz, Nathaniel R. Greenbaum, Matthew P. Lungren, Chih-ying Deng, Roger G. Mark, Steven Horng

**Affiliations:** 1 Institute of Medical Engineering & Science, Massachusetts Institute of Technology, Cambridge, MA USA; 2 Department of Radiology, Beth Israel Deaconess Medical Center, Boston, MA USA; 3 Department of Emergency Medicine, Beth Israel Deaconess Medical Center, Boston, MA USA; 4 Department of Radiology, Stanford University, Palo Alto, CA USA; 5 Department of Biomedical Informatics, Harvard Medical School, Boston, MA USA

**Keywords:** Translational research, Radiography

## Abstract

Chest radiography is an extremely powerful imaging modality, allowing for a detailed inspection of a patient’s chest, but requires specialized training for proper interpretation. With the advent of high performance general purpose computer vision algorithms, the accurate automated analysis of chest radiographs is becoming increasingly of interest to researchers. Here we describe MIMIC-CXR, a large dataset of 227,835 imaging studies for 65,379 patients presenting to the Beth Israel Deaconess Medical Center Emergency Department between 2011–2016. Each imaging study can contain one or more images, usually a frontal view and a lateral view. A total of 377,110 images are available in the dataset. Studies are made available with a semi-structured free-text radiology report that describes the radiological findings of the images, written by a practicing radiologist contemporaneously during routine clinical care. All images and reports have been de-identified to protect patient privacy. The dataset is made freely available to facilitate and encourage a wide range of research in computer vision, natural language processing, and clinical data mining.

## Abstract (summary)

**Table:** 

Measurement(s)	Radiograph • investigation results report
Technology Type(s)	Chest Radiography • digital curation • Radiologist
Sample Characteristic - Organism	Homo sapiens

Machine-accessible metadata file describing the reported data: 10.6084/m9.figshare.10303823

## Background & Summary

Chest radiography is a routinely used imaging modality to assess the chest and is the most common medical imaging study in the world. Chest radiographs are used to identify acute and chronic cardiopulmonary conditions, verify that devices such as pacemakers, central lines, chest tubes, and gastric tubes are correctly positioned, and to assist in related medical workups. In the U.S., the number of radiologists as a percentage of the physician workforce is decreasing^1^, and the geographic distribution of radiologists favors larger, more urban counties^2^. Even when trained radiologists are available, chest radiographs are often interpreted first by non-radiologists such as intensivists and emergency physicians, before being overread by a radiologist. Delays and backlogs in timely medical imaging interpretation have demonstrably reduced care quality in such large health organizations as the U.K. National Health Service^3^ and the U.S. Department of Veterans Affairs^4^. The situation is even worse in resource-poor areas, where radiology services are extremely scarce. As of 2015, only 11 radiologists served the 12 million people of Rwanda^5^, while the entire country of Liberia, with a population of four million, had only two practicing radiologists^6^. Accurate automated analysis of radiographs has the potential to improve the efficiency of radiologist workflow and extend expertise to under-served regions.

The combination of burgeoning datasets with increasingly sophisticated algorithms has resulted in a number of significant advances in other application areas of computer vision^7,8^. A key requirement in the application of these advances to automated chest radiograph analysis is a sufficiently large data set that allows competing algorithms from different groups to be directly compared to one another. Here we present MIMIC Chest X-ray (MIMIC-CXR), a large publicly available dataset of chest radiographs with free-text radiology reports. The dataset contains 377,110 images corresponding to 227,835 radiographic studies performed at the Beth Israel Deaconess Medical Center (BIDMC) in Boston, MA. The dataset is de-identified to satisfy the US Health Insurance Portability and Accountability Act of 1996 (HIPAA) Safe Harbor requirements. Protected health information (PHI) has been removed. Randomly generated identifiers are used to group distinct reports and patients. The dataset is intended to support a wide body of research in medicine including image understanding, natural language processing, and decision support.

## Methods

The creation of MIMIC-CXR required handling three distinct data modalities: electronic health record data, images (chest radiographs), and natural language (free-text reports). These three modalities were processed independently and ultimately combined to create the database. The project was approved by the Institutional Review Board of BIDMC (Boston, MA). Requirement for individual patient consent was waived because the project did not impact clinical care and all protected health information was removed.

### Electronic health record

The BIDMC operates a locally built electronic health record (EHR), including a Radiology Information System (RIS), to store and process clinical and radiographic data. We identified the set of chest radiographs to include in a two stage process. First, we identified a cohort of patients. We queried the BIDMC EHR for chest radiograph studies acquired in the emergency department between 2011–2016, and extracted *only* the set of patient identifiers associated with these studies. A collection of images associated with a single report is referred to as a study, identified by a unique identifier, the study ID. We then extracted all chest radiographs and radiology reports available in the RIS for this set of patients between 2011–2016.

For anonymization purposes, two sets of random identifiers were generated. First, a random identifier was generated for each patient in the range 10,000,000–19,999,999, which we refer to as the subject_id. Each patient was also assigned a date shift which mapped their first index admission year to a year between 2100–2200. This ensures anonymity of the data while preserving the relative temporality of patient information, which is crucial for appropriate processing of the data. Note that this date shift removes all information about seasonality or day of the week for the studies. Second, as each report will be associated with a single unique study identifier, we generated a random identifier for each study in the range 50,000,000–59,999,999. We refer to the anonymized study identifier as the study_id. As multiple images may be associated with the same study (e.g. one frontal and one lateral image), multiple images in MIMIC-CXR have the same study_id.

### Chest radiographs

Chest radiographs were sourced from the hospital picture archiving and communication system (PACS) in Digital Imaging and Communications in Medicine (DICOM) format. DICOM is a common format which facilitates interoperability between medical imaging devices. The DICOM format contains meta-data associated with one or more images, and the DICOM standard stipulates strict rules around the structure of this information. The DICOM standard is updated regularly each year. In this work, we use the DICOM Standard version 2017e^9^. This standard is available online: http://dicom.nema.org/medical/dicom/2017e/. Although it was not necessary to retain images in DICOM format for MIMIC-CXR, we believe it is beneficial for the data to be distributed in a standard format used in hospitals worldwide. Retaining data in DICOM format encourages the development of algorithms which can process data in this format and, consequently, are more readily applicable to real clinical practice.

The acquired DICOM images contained PHI which required removal for conformance with HIPAA. PHI was removed from both the DICOM meta-data and the pixel values. DICOM meta-data is organized into a finite set of data elements; each element is uniquely identified by a numerical tag and stores a fixed concept. The DICOM standard (Table E.1-1, PS 3.15) describes a number of de-identification profiles, each of which outlining data elements which must be removed or modified in order to anonymize patient data^9^. We followed the Basic Application Level Confidentiality Profile with the Clean Descriptors Option, Retain Longitudinal Temporal Information Modified Dates, Clean Pixel Data, and Clean Graphics options. The De-identification Method Code Sequence (0012,0064) was set to a length five sequence codifying the aforementioned profile choices, and Patient Identity Removed (0012, 0062) was set to “YES” for all images. Broadly, this de-identification strategy represents our intent to fully remove individual patient information while retaining useful parameters stored in descriptive attributes and preserving the chronology of radiographs for a single patient.

#### Basic application level confidentiality profile

DICOMs were initially de-identified using Orthanc v1.0.0^10^. As the unique identifier for the image is considered PHI, Orthanc generated new unique identifiers for each image, and this identifier is used as the filename for each image. The DICOM standard requires that a unique object identifier (UID) be generated for the study, series, and instance (in our case, the image). These UIDs were generated using the Universally Unique Identifier (UUID) approach and stored in the DICOM header as “2.25.” followed by an integer up to 39 digits in length. Anonymized identifiers for the patient and study were inserted into the patient and study DICOM data elements.

#### Clean descriptors option

A number of data elements which contained unstructured text were present. Data elements which provided no scientific value were removed. Data elements which were useful but contained free-text were homogenized using manually curated look up tables. Text which was not present in the data element look up table was removed.

#### Retain longitudinal temporal information modified dates

Longitudinal information is important for contextualizing sequential chest radiographs for an individual patient. Temporal information was retained by shifting dates relating to patient care using the patient-specific date shift previously generated. Dates stored in the data elements listed in Table E.1-1 under the Retain Longitudinal Temporal Information Modified Dates were shifted using this methodology^9^. The time of day was not modified by the date shifting process. Notably, the ontology used for certain fields is versioned using a date (e.g. SNOMED), and these dates were retained without a date shift.

#### Clean pixel data

After processing of DICOM meta-data, de-identification of the image pixel values was necessary. Images sometimes contain “burned in” annotations: areas where pixel values have been modified after image acquisition in order to display text. These annotations usually contain information necessary for image interpretation, such as image orientation, anatomical position of the subject, and timestamp of image capture. However, annotations may also include patient names, medical record numbers, and patient date of birth. The resulting image, with textual annotations encoded within the pixel themselves, is then transferred from the modality to PACS. Since the annotations are applied at the modality level, it is impossible to recover the original image without annotations. Although these annotations would be more effectively encoded as an overlay rather than in the pixels themselves, burned in annotations are still a common practice in medicine.

Images were de-identified using a custom algorithm which removed dates and patient identifiers, but retained radiologically relevant information such as orientation. The algorithm applied an ensemble of image preprocessing and optical character recognition approaches to detect text within an image. Images were binarized to enhance contrast of the text with the background. Three thresholds were used to binarize the image: one based upon the maximum pixel intensity, one based upon the minimum pixel intensity, and one fixed to a specific pixel value frequently used by the modality when adding text. Optical character recognition was performed using the tesseract library v3.05.02^11^. In the DICOM header, the data element Burned In Annotation (0028, 0301) was set to “YES” if any text was detected in the image pixels, otherwise it was set to “NO”.

Text was classified as PHI using a set of custom regular expressions which aimed to be conservative in removal of text and allow for errors in the optical character recognition. If a body of text was suspected to be PHI, all pixel values in a bounding box encompassing the PHI were set to black.

#### Clean graphics

Table E.1-1 of the DICOM standard lists a set of standard data elements which store overlay planes, graphics, or annotations and must be cleaned of PHI when using the Clean Graphics option^9^. These data elements were removed when present. Subsequently, the Graphic Object Sequence (0070, 0009) under the Graphic Annotation Sequence (0070, 0001) data element was used to store coordinates of the PHI obscuring black boxes used to clean the pixel data.

#### Validation of de-identification

All unique DICOM metadata attribute values were manually reviewed and did not contain PHI. We then manually reviewed the pixel data for 6,900 radiographs. Each image was reviewed by two independent annotators. 180 images were identified for a secondary consensus review; none of which ultimately had PHI. The most common causes for annotators to request consensus review were: (1) existence of a support device such as a pacemaker, (2) text identifying in-hospital location (e.g. “MICU”), and (3) obscure text relating to radiograph technique (e.g. “prt rr slot 11”).

### Radiology reports

During routine care, radiologists have access to brief text summarizing the underlying medical condition, the reason for examination, and prior imaging studies performed. The PACS workstation used by clinicans to view images allows for dynamic adjustment of the mapping between pixel value and grey-level display (“windowing”), side-by-side comparison with previous imaging, overlaying of patient demographics, and overlaying of imaging technique. Reports are transcribed during reading of an image series using a real-time computer voice recognition service.

Radiology reports for all identified studies were extracted from the underlying EHR. Reports were exported in eXtensible Markup Language (XML). Text was extracted from the XML file and organized into single text files denoted by the study identifier. Reports are archived with linebreaks to ensure individual lines are no longer than 79 characters, and contain up to four segments delimited by underscores repeated to the width of the page. The four segments are: an optional addendum, a report header with patient information, clinical information imported from the EHR, and the main body of the report. Only the addendum and main segments of the report are written by the radiologist.

The free-text portion of each report was processed as follows. First, the header segment was removed, as it contained administrative information that is not necessary for interpretation of the report. The clinical information segment was also removed from the textual report. Electronic signatures located at the end of the report were identified using a set of regular expressions and removed. As a result, reports have a maximum of two segments: an optional addendum, and the body of the report. The free-text report was then de-identified using a rule-based approach based upon prior work^12–14^. PHI of any length was consistently replaced with three underscores (“_ _ _”). Study reports are stored in individual text files named using the anonymous study identifier.

We evaluated the performance of our de-identification approach by manually annotating 2,238 radiology reports for PHI. We annotated all text specified by HIPAA as PHI. Furthermore, we annotated large locations (such as U.S. states and countries), years, and clinical provider names as PHI. These are not required to be removed by HIPAA, but we have elected to remove these entities. We compared the performance of our automated annotation method against this gold standard set. Each document contained an average of 642 characters and 145 “tokens” (words), with 324,641 tokens across the entire set. Of these tokens, 9,778 were considered PHI, with an average of 4.4 PHI tokens per document. Our approach did not detect 8 of the 9,778 PHI tokens. The eight tokens consisted of: three provider names which were also English dictionary words (e.g. Rose), one misspelled provider name, two dates with typographical errors, and two sets of initials for provider names. We manually removed these entities from the final dataset.

Each report was also associated with meta-data in the source XML file, including the following fields: patient identifier, study identifier, date and time of study, and the examination name. Examination names are standardized within the BIDMC under the Procedure Code Sequence (0008, 1032) using Simon-Leeming codes^15,16^. Simon-Leeming codes are referenced as “CLP” in the standard set of code systems described by the Health Level 7 (HL7) organization (HL7 v2 Table 0396, https://www.hl7.org/special/committees/vocab/table_0396/index.cfm). Table 1 lists the number of studies for the top ten most frequent examination names. An additional 14 examination names are present with very few (<60) studies for each, for a total of 24 distinct examination names.

**Table 1:** Number of DICOM images for the top ten most frequent radiological examination types.

Code	Examination name	DICOMs	(%)
C11	CHEST (PA AND LAT)	248,664	65.94
C12	CHEST (PORTABLE AP)	126,292	33.49
PC111	DX CHEST PORTABLE PICC LINE PLACEMENT	329	0.09
PC171	DX CHEST PORT LINE/TUBE PLCMT 1 EXAM	255	0.07
PC172	DX CHEST PORT LINE/TUBE PLCMT 2 EXAMS	165	0.04
PC173	DX CHEST PORT LINE/TUBE PLCMT 3 EXAMS	157	0.04
PC3	DX CHEST & RIBS	131	0.03
PC1	DX CHEST WITH DECUB	104	0.03
C13	CHEST (SINGLE VIEW)	85	0.02
PC113	DX CHEST 2 VIEW PICC LINE PLACEMENT	77	0.02

Examination type is classified using Simon-Leeming codes^35^, and is available in the DICOM header within the Procedure Code Sequence (0008, 1032).

## Data Records

All data are made available on PhysioNet^17^. Access is controlled, requiring user registration, completion of a credentialing process, and signing of a data use agreement (see usage notes). The MIMIC-CXR project page on PhysioNet describes the dataset and informs users how they may apply for access^18^.

Images and reports are organized into subfolders named according to the anonymous patient identifier. Each patient subfolder contains a single folder and a single text file for each imaging study made available for that patient.

### Study folder

Folder names are set to the anonymized study identifier prefixed with the letter “s” (e.g. s########). This study folder contains all imaging performed for the study in DICOM format^9^; namely one or more chest radiographs.

#### Study text

The free-text radiology report is made available in a plain text file. The stem of the file has the same filename as the study folder, and it can be differentiated from the study folder by its extension (“.txt”).

An example of this layout is provided in Table 2. All patient identifiers begin with the digit 1 and have a total length of 8 digits. All study identifiers begin with the digit 5 and have a total length of 8 digits. DICOM file names are unique 40 character hexadecimal strings with dashes separating groups of eight characters.

**Table 2:** Example of the data record layout using patient 10000032.

Level 1	Level 2	Description
s50414267.txt		Radiology report.
s50414267		Study folder.
	02aa804e-bde0afdd-112c0b34-7bc16630-4e384014.dcm	DICOM file.
	174413ec-4ec4c1f7-34ea26b7-c5f994f8-79ef1962.dcm	DICOM file.
s53189527.txt		Radiology report.
s53189527		Study folder.
	2a2277a9-b0ded155-c0de8eb9-c124d10e-82c5caab.dcm	DICOM file.
	e084de3b-be89b11e-20fe3f9f-9c8d8dfe-4cfd202c.dcm	DICOM file.
s53911762.txt		Radiology report.
s53911762		Study folder.
	68b5c4b1-227d0485-9cc38c3f-7b84ab51-4b472714.dcm	DICOM file.
	fffabebf-74fd3a1f-673b6b41-96ec0ac9-2ab69818.dcm	DICOM file.
s56699142.txt	Radiology report.	
s56699142		Study folder.
	ea030e7a-2e3b1346-bc518786-7a8fd698-f673b44c.dcm	DICOM file.

All records listed in this table are located within the *p10000032* folder. Column headers indicate the depth level in the folder hierarchy. Level 1 folders and files are present within the *p10000032* folder. Level 2 files are present in the subfolder immediately above.

To alleviate issues many software packages have with parsing a large number of files in a single folder, we group patient folders into higher level folders based on the first three characters of the folder name. For example, the folder “p10000032” would be placed in the higher level folder “p10”, “p11000011” would be placed in “p11”, and so on.

A mapping file is provided which lists all image names with the corresponding study identifier and patient identifier.

**Fig. 1:**
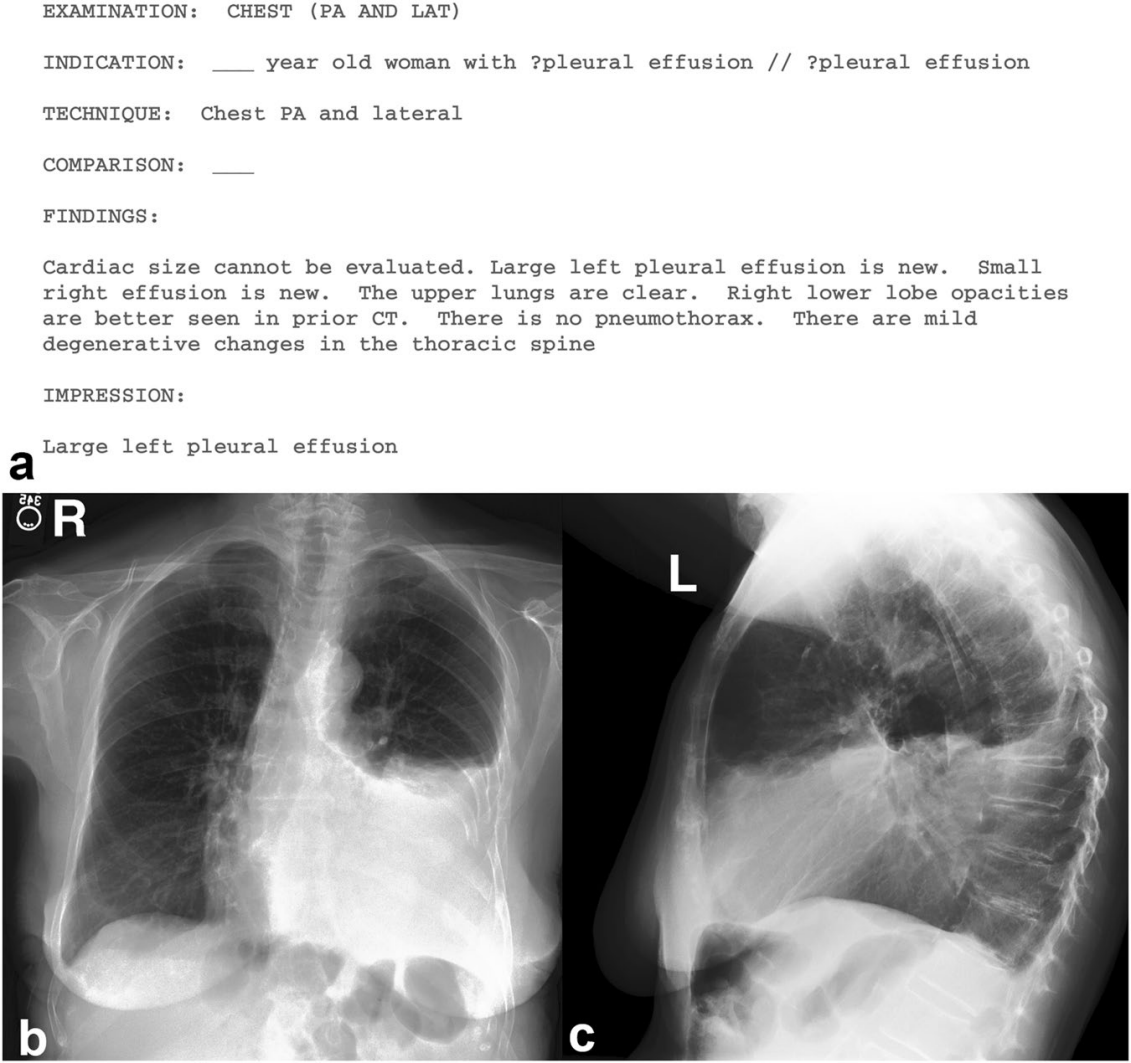
Example study contained in MIMIC-CXR. Above (**a**), the radiology report provides the interpretation of the image. PHI has been removed and replaced with three underscores (_ _ _). Below, the two chest radiographs for this study are shown: (**b**) the frontal view (left image) and (**c**) the lateral view (right image).

An example study is provided in Fig. 1.

## Technical Validation

Creation of MIMIC-CXR followed good software practices, including source code control, continuous integration, and test-driven development^19^. DICOM meta-data was parsed to determine the anatomy examined and view position, and a small number of mislabeled radiographs which were not examining the chest were removed (e.g. pelvic and abdominal radiographs). We validated DICOM meta-data consistency with the DICOM standard using the open-source DICOM validator *dciodvfy*, available from https://www.dclunie.com/dicom3tools/dciodvfy.html.

**Fig. 2:**
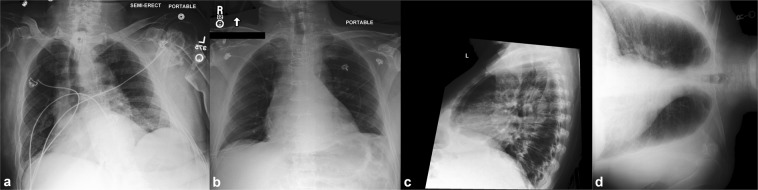
Images which highlight the amount of variation present in MIMIC-CXR. From left to right: (**a**) poor patient positioning, (**b**) black box obscuring potential PHI, (**c**) secondary collimation to improve image quality, and (**d**) incorrect image orientation information in the meta-data.

Aside from view parsing and de-identification of the dataset, no filtering or processing of the images was performed. Consequently, images exhibit a number of phenomena common in daily practice. The quality of images varies, both in terms of technique and in terms of patient positioning (e.g. not all patients are healthy enough to stand for a posterior-anterior radiograph, or sit upright for an anterior-posterior radiograph). Images may unintentionally omit anatomy present in a standard chest radiograph, or have objects that obstruct important anatomy. Finally, collimation can also be applied at the modality to crop the image, improving post-processing in the area of interest. Figure 2 presents a selection of images from the dataset exhibiting challenges to automated processing. It is important for methods using the database to be capable of accommodating these variations as they routinely occur in clinical practice.

## Usage Notes

Use of the data requires proof of completion of a course on human subjects research (e.g. from the Collaborative Institutional Training Initiative^20^). Data access also requires signing of a data use agreement that stipulates, among other items, that the user will not share the data, will not attempt to re-identify any patients or institutions, and will release code associated with any publication using the data, as is the process for MIMIC-III^21^ and eICU-CRD^22^. Once approved, data can be directly downloaded from the MIMIC-CXR Database project on PhysioNet.

We have provided publicly accessible Jupyter Notebooks^23,24^ to demonstrate usage of the data. These notebooks supplement online documentation and provide commentary on best practices when working with the data. These notebooks are made available in an openly available code repository^25^. One notebook provided reproduces all tables in this paper from the publicly available dataset.

A core aim in publicly releasing MIMIC-CXR is to foster collaboration in medical image processing and secondary analysis of electronic health records. We believe that publicly accessible code using publicly available data will accelerate research in the field and ensure reproducibility of future studies^26,27^. We hope that users will contribute code used in their studies to this repository or link to their own repositories of openly available code to ensure maximum visibility in the community. Online documentation is made available and will be updated as needed: https://mimic-cxr.mit.edu.

### Related datasets

While MIMIC-CXR can support a large body of research independently, a number of other datasets exist which may compliment future work. The Japanese Society of Radiological Technology (JSRT) Database contains 247 images with labels of chest nodules as confirmed by subsequent computed tomography (CT)^28^. Notably, the dataset is provided with annotations segmenting the lungs and heart. The Montgomery County chest X-ray set (MC) and the Shenzhen Hospital X-ray set (SH) are publicly available datasets focusing on classification of tuberculosis^29,30^. The MC dataset, courtesy of the National Library of Medicine, contains 138 posterior-anterior chest x-rays in DICOM format of which 58 contain radiographic findings associated with tuberculosis. The SH dataset, courtesy of the Shenzhen No. 3 People’s Hospital, contains 662 posterior-anterior chest x-rays in PNG format of which 336 display manifestations of tuberculosis. The Open-I Indiana University Chest X-ray dataset contains 8,121 images associated with 3,996 de-identified radiology reports^31^. The NIH released ChestX-ray14 (originally ChestX-ray8), a collection of 112,120 frontal chest radiographs from 30,805 distinct patients with 14 binary labels indicating existence pathology or lack of pathology^32^. A total of 224,316 chest radiographs for 65,240 patients admitted to Stanford Hospital were released with the CheXpert labeler by researchers at Stanford University^33^. These images were released with 14 labels derived from automatic processing of the notes in a similar fashion to the NIH dataset, though the labels do not perfectly overlap, and the CheXpert labels include an additional “uncertain” category. Similarly the University of Alicante released a set of 160,868 chest radiographs from 69,882 patients admitted to the Hospital San Juan de Alicante, Spain^34^. This dataset was released with structured labels for each radiograph acquired either manually or automatically. Manual labels were sourced from the reports associated with over 20,000 images. Automated labels for the remaining images were generated by a neural network classifier trained using the aforementioned manual labels. Notably, there were 297 label categories, and the aim of the label set was to exhaustively cover all radiographic, anatomic, and diagnostic labels mentioned in the report.

## Data Availability

Due to the use of real patient information during the de-identification process, the code used to prepare the dataset cannot be made publicly available. Example usage code, including loading a DICOM study and linking it with its associated free-text radiology report, has been made available publicly^25^.
